# Human Tooth as a Fungal Niche: Candida albicans Traits in Dental Plaque Isolates

**DOI:** 10.1128/mbio.02769-22

**Published:** 2023-01-05

**Authors:** Zhenting Xiang, Rohan S. Wakade, Apoena Aguiar Ribeiro, Weiming Hu, Kyle Bittinger, Aurea Simon-Soro, Dongyeop Kim, Jiyao Li, Damian J. Krysan, Yuan Liu, Hyun Koo

**Affiliations:** a Biofilm Research Laboratories, Department of Orthodontics, Divisions of Community Oral Health & Pediatric Dentistry, School of Dental Medicine, University of Pennsylvania, Philadelphia, Pennsylvania, USA; b State Key Laboratory of Oral Diseases and National Clinical Research Center for Oral Diseases, Department of Cariology and Endodontics, West China Hospital of Stomatology, Sichuan University, Chengdu, China; c Department of Pediatrics, Carver College of Medicine, University of Iowa, Iowa City, Iowa, USA; d Division of Diagnostic Sciences, Adams School of Dentistry, University of North Carolina at Chapel Hill, Chapel Hill, North Carolina, USA; e Division of Gastroenterology, Hepatology, and Nutrition, Children's Hospital of Philadelphia, Philadelphia, Pennsylvania, USA; f Department of Stomatology, School of Dentistry, University of Seville, Seville, Spain; g Department of Preventive Dentistry, School of Dentistry, Jeonbuk National University, Jeonju, South Korea; h Department of Microbiology and Immunology, Carver College of Medicine, University of Iowa, Iowa City, Iowa, USA; i Department of Preventive & Restorative Sciences, School of Dental Medicine, University of Pennsylvania, Philadelphia, Pennsylvania, USA; j Center for Innovation & Precision Dentistry, School of Dental Medicine and School of Engineering & Applied Sciences, University of Pennsylvania, Philadelphia, Pennsylvania, USA; Georgia Institute of Technology

**Keywords:** biofilms, *Candida albicans*, streptococci, clinical isolates, childhood caries, interkingdom interactions *Streptococcus*, dental caries

## Abstract

Candida albicans, a fungus typically found in the mucosal niche, is frequently detected in biofilms formed on teeth (dental plaque) of toddlers with severe childhood caries, a global public health problem that causes rampant tooth decay. However, knowledge about fungal traits on the tooth surface remains limited. Here, we assess the phylogeny, phenotype, and interkingdom interactions of C. albicans isolated from plaque of diseased toddlers and compare their properties to reference strains, including 529L (mucosal isolate). C. albicans isolates exhibit broad phenotypic variations, but all display cariogenic traits, including high proteinase activity, acidogenicity, and acid tolerance. Unexpectedly, we find distinctive variations in filamentous growth, ranging from hyphal defective to hyperfilamentous. We then investigate the ability of tooth isolates to form interkingdom biofilms with Streptococcus mutans (cariogenic partner) and Streptococcus gordonii (mucosal partner). The hyphal-defective isolate lacks cobinding with S. gordonii, but all C. albicans isolates develop robust biofilms with S. mutans irrespective of their filamentation state. Moreover, either type of C. albicans (hyphae defective or hyperfilamentous) enhances sucrose metabolism and biofilm acidogenicity, creating highly acidic environmental pH (<5.5). Notably, C. albicans isolates show altered transcriptomes associated with pH, adhesion, and cell wall composition (versus reference strains), further supporting niche-associated traits. Our data reveal that C. albicans displays distinctive adaptive mechanisms on the tooth surface and develops interactions with pathogenic bacteria while creating an acidogenic state regardless of fungal morphology, contrasting with interkingdom partnerships in mucosal infections. Human tooth may provide new insights into fungal colonization/adaptation, interkingdom biofilms, and contributions to disease pathogenesis.

## INTRODUCTION

Candida albicans often colonizes mucosal surfaces, including the gastrointestinal tract, reproductive tract, and oral cavity, where it can be part of the commensal microbiota in the healthy population (1, 2). However, C. albicans becomes virulent under pathological circumstances, such as immune deficiencies, environmental changes, and microbiota imbalance, or when infecting implanted surfaces, causing localized or systemic infections (3, 4). The oral cavity provides a uniquely complex ecosystem for fungal colonization due to its anatomical features comprised of soft (mucosal) and mineralized hard (tooth) tissues as well as presence of abiotic (denture/implants) surfaces (5). C. albicans has been traditionally associated with oral mucosa infection rather than teeth (6, 7). However, the tooth surface has been increasingly recognized as an important niche for *Candida* colonization and dental biofilm (plaque) formation with bacterial species (8).

Results from several clinical studies revealed that *Candida* (especially C. albicans) is frequently detected in dental plaque from children with severe early childhood caries (S-ECC), a rampant and painful tooth decay with systemic complications affecting hundreds of millions of toddlers worldwide (9–11). In particular, the presence of C. albicans is associated with caries severity evidenced by both experimental and clinical studies, indicating that C. albicans plays a distinct role in the pathogenesis of S-ECC (12, 13). Furthermore, microbiome studies show a positive correlation between the abundance of C. albicans and the presence of Streptococcus mutans, a cariogenic bacterial pathogen, suggesting interkingdom interactions in the dental plaque (10, 11). Notably, when C. albicans was coinfected with S. mutans in an *in vivo* caries model, they synergistically enhanced microbial colonization on teeth and induced the development of rampant caries lesions (7, 14).

Laboratory studies have demonstrated that C. albicans interacts with S. mutans through biochemical, metabolic, and physical mechanisms, resulting in the formation of highly cariogenic interkingdom biofilms (15–19). However, previous work was mainly obtained from investigations using C. albicans laboratory reference strains originally isolated from the bloodstream or mucosa (20, 21), which may have genetic and phenotypic differences with clinical isolates from diseased children with S-ECC (22–24). Thus, understanding the pathobiology of C. albicans isolated from tooth surfaces and how it differs from reference strains could provide important insights into the role of C. albicans in interkingdom interactions associated with this devastating childhood disease.

Here, we analyzed the genotype, phenotype, and interkingdom interactions of C. albicans strains isolated from dental plaque of children affected by S-ECC. The tooth isolates exhibited interesting variations in hyphal growth, ranging from hyphae defective to hyperfilamentous. Yet all isolates shared common cariogenic traits, including acidogenesis and the ability to develop metabolic interactions with S. mutans and form interkingdom biofilms. These properties shared by C. albicans tooth isolates promoted highly acidogenic biofilms capable of lowering the pH of saliva to pathological levels (pH < 5.5) regardless of hyphal formation. Notably, we found that tooth isolates were transcriptionally distinct from the reference strains while modulating the expression of pH- and adhesion/cell wall-related genes when grown with S. mutans in interkingdom biofilms. Our results reveal that C. albicans on tooth surface develops biofilms and influences virulence through distinctive mechanisms, including hyphal form variations, highlighting the remarkable plasticity of C. albicans to adapt to a distinct habitat and promote a disease-causing state.

## RESULTS

### Phylogenetic analysis of C. albicans isolates.

C. albicans strains were isolated from S-ECC plaque samples, and multilocus sequence typing (MLST) was performed for their genotypic characterization. DNA sequencing of fragments from the coding region of seven housekeeping genes generated a data set of 2,883 bp for each isolate (see Table S1 in the supplemental material). The analysis yielded 15 distinct diploid sequence types (DSTs) from 16 isolates (DST277 was shared by isolates UR12 and UR15), among which 12 DSTs were newly identified in the current study. We identified a total of 11 new sequence types (STs) in the seven loci (ST184 for *AAT1a*; ST118, -119, and -120 for *AAC1*; ST174 for *ADP1*; ST177 and -178 for *MPIb*; ST241 and -243 for *SYA1*; and ST311 and -312 for *VPS13*). These new STs and DSTs were submitted to the PubMLST database (https://pubmlst.org/organisms/candida-albicans).

10.1128/mbio.02769-22.1 TABLE S1 ST assignments at each of the seven MLST loci for the tooth isolates. ST numbers in red represent new STs or DSTs identified in the current study. Download Table S1, XLSX file, 0.01 MB. Copyright © 2023 Xiang et al. 2023 Xiang et al. https://creativecommons.org/licenses/by/4.0/ This content is distributed under the terms of the Creative Commons Attribution 4.0 International license.

**FIG 1:**
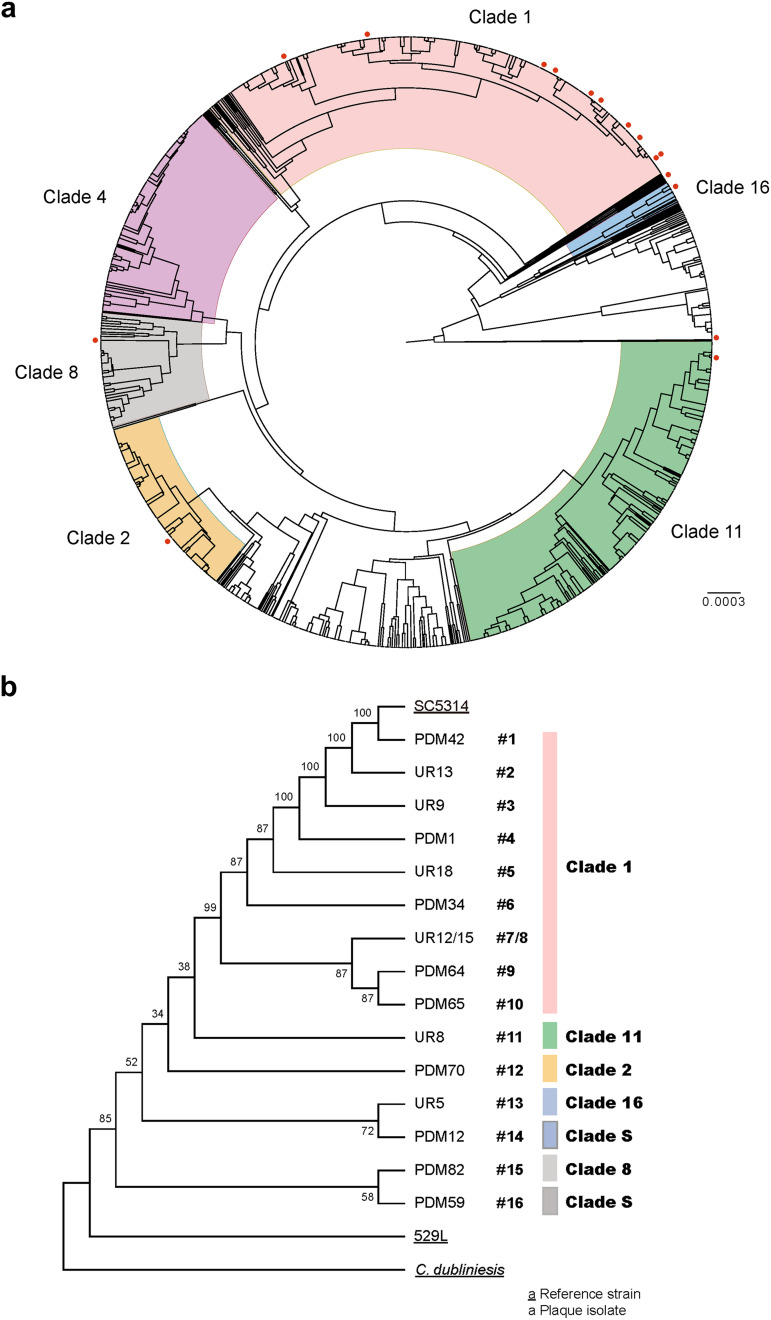
Phylogenetic analyses of C. albicans isolates. (a) The phylogram was constructed from UPGMA analysis of 16 isolates in this study and a global collection of 1,491 isolates retrieved from the MLST database using uncorrected *P* distances based on MLST of the 7 loci. The major clades of global C. albicans are indicated, and our isolates are labeled as red dots to show their phylogenetic distribution. (b) Phylogenetic relationship of C. albicans isolates from S-ECC children’s plaque samples based on MLST. Clades were marked by different colors according to the phylogenetical analysis, and each isolate was assigned to a number shown on the right side of the dendrogram, respectively.

We combined our isolates sequences with those of 1,491 strains deposited in the PubMLST database to further analyze the evolutionary relationships between the isolates. C. albicans SC5314 (isolated from human blood), C. albicans 529L (isolated from the oral mucosa of a patient with oral candidiasis), and Candida dubliniensis ATCC MYA-646 were included as reference strains. As shown in Fig. 1a, we placed 14 isolates into 5 previously defined clades, clade 1, clade 2, clade 8, clade 11, and clade 16. The remaining two isolates (ST3679 and ST3680) were singletons. The greatest number of isolates (10 out of 16) were found in clade 1 (62.5%), which is the most prevalent clade globally (25). We renumbered the isolates according to the genetic relationships (Fig. 1b). The phylogenetic linkages between the strains indicated that the isolates were more closely associated with reference strain C. albicans SC5314 than the mucosal strain 529L, and C. dubliniensis was distinct from all C. albicans isolates.

### Phenotypic profiling of C. albicans isolates.

The phenotypic plasticity of C. albicans has been associated with its ability to adapt to and colonize a variety of niches in the human host (1, 26). However, the fungal traits on the tooth surface (a unique mineralized hard tissue) remain poorly explored. By monitoring the growth, we found that all the tooth isolates were able to proliferate under conditions that mimic the oral cavity environment (37°C, pH 7.0) (Fig. S1a and b). We further performed comprehensive analyses to explore the phenotypic differences of the C. albicans isolates under different stressors, including temperature, oxidative stress, and antifungal agent.

10.1128/mbio.02769-22.3 FIG S1 Growth characteristic of C. albicans strains. (a) Growth curve in UFTYE at 37°C, pH 7.0. (b) Total viable counts (CFU) of C. albicans at OD of 0.8. Download FIG S1, TIF file, 2.0 MB. Copyright © 2023 Xiang et al. 2023 Xiang et al. https://creativecommons.org/licenses/by/4.0/ This content is distributed under the terms of the Creative Commons Attribution 4.0 International license.

**FIG 2:**
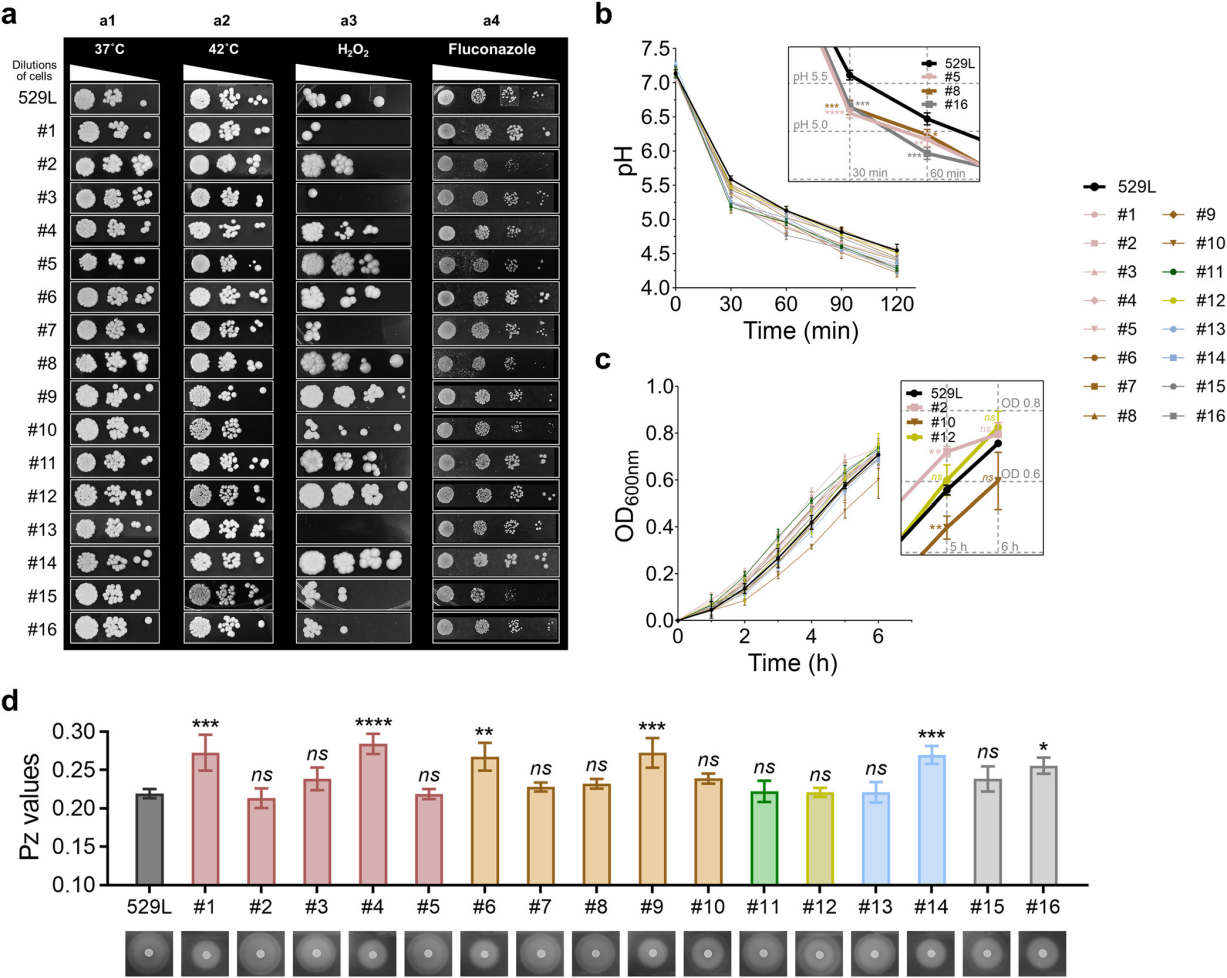
Phenotypic profiling of C. albicans isolates. (a) Stress tolerance. Serial dilutions of C. albicans cells were spotted on the plates with regular growth conditions (37°C, YPD agar), thermal stress (42°C, YPD agar), oxidative stress (YPD agar containing 10 mM H_2_O_2_), and antifungal agents (YPD agar containing 2 μg/mL fluconazole). (b) Glycolytic acid production determined by the decrease in pH of the solution containing glucose over a period of 120 min. (c) Aciduric ability assay. Data are presented as growth curve of C. albicans isolates cultured in ultrafiltered tryptone-yeast extract broth (UFTYE; pH 4.0). (d) Secreted aspartyl protease activity. The enzyme activity was determined by the agar plate method (bottom). *Pz* values were calculated according to the diameters of the colony and precipitation zone on the plate. Data are presented as mean ± SD of *n* = 3 independent replicates. Values are significantly different from 529L (reference strain) at *, *P < *0.05; **, *P < *0.01; ***, *P < *0.001; and ****, *P < *0.0001, while *ns* indicates that the values are not significantly different from 529L.

All the isolates exhibited similar growth patterns under heat stress (Fig. 2a, panels a1 and a2). As for the oxidative stress test, isolates 5, 9, 11, 12, and 14 exhibited higher resistance to hydrogen peroxide (10 mM) than 529L, while isolates 1, 3, 7, and 13 were more susceptible to oxidative stress; in particular, isolate 13 showed no growth at any cell density (Fig. 2a, panel a3). We also conducted a susceptibility test using fluconazole as an antifungal stressor. As shown in Fig. 2a, panel a4, isolates 2, 4, 8, and 15 showed reduced growth, with smaller colonies formed on the yeast extract-peptone-dextrose (YPD) plate containing fluconazole (2 μg/mL) than other isolates, indicating a higher susceptibility to the antifungal stress. However, we did not observe any clade-specific trends regarding the strain’s susceptibility to stressors, while there was no significant association between the phenotypes and cluster designation. Altogether, our results showed that the C. albicans isolates varied widely in their response to various stressors without a specific phenotype.

### Cariogenic trait characterization of C. albicans isolates.

Cariogenicity is associated with the creation of acidic pH milieu in biofilms on the tooth surface. C. albicans can produce and tolerate acids, which are essential factors for caries development (27). We therefore performed a glycolytic pH drop assay to assess acid-producing and acid tolerance properties of the tooth isolates. In this assay, microbial cells rapidly degrade glucose and lower the pH value of the suspension until they can no longer maintain a cytoplasmatic pH compatible with glycolysis. Thus, the rate of pH drop reflects the acidogenic capacity of the cells, while the final pH values reflect acid tolerance (28). As shown in Fig. 2b, all isolates rapidly acidified the suspension within 30 min when glucose was added at a final concentration of 1%, and the pH was lowered to or below 5.5, which is the critical pH for enamel acid dissolution (29). Among them, isolates 5, 8, and 16 (Fig. 2b, inset) showed the lowest pH value at the early time points (30 min and 60 min), indicating more and faster acid production than other isolates. Interestingly, the pH values by the reference strain 529L were higher across all time points, suggesting lower acidogenicity and acid tolerance than the isolates. We further tested their survival at pH 4.0 (a highly acidic pH value typically found in cariogenic biofilms) and observed that all isolates grew continuously (Fig. 2c), indicating adaptation in acidic microenvironments.

The ability of C. albicans to produce proteolytic enzymes, such as secreted aspartyl proteinases (Saps), which facilitates adhesion and promotes degradation of dentin via collagenolysis, is another important factor in caries progression (30). Hence, the enzymatic activity of Saps was assessed by measuring the levels of proteolysis around the C. albicans colonies on agar plates. As shown in Fig. 2d, a distinct zone of proteolysis (opaque halo) around the colony could be seen in all C. albicans isolates, indicating positive Sap activities. Furthermore, we calculated the *Pz* value (i.e., the ratio of colony diameter to colony-and-halo diameter) for each C. albicans isolate; the values ranged from 0.21 to 0.28, indicating high levels of Sap activity (values lower than 0.64) according to the correlation between *Pz* value and Sap activity as standardized in reference 31. Thus, all C. albicans isolates show common traits of strong acidogenicity, aciduricity, and high proteolytic activity, which are associated with dental caries pathogenesis.

### Hyphal morphogenesis of C. albicans isolates.

**FIG 3:**
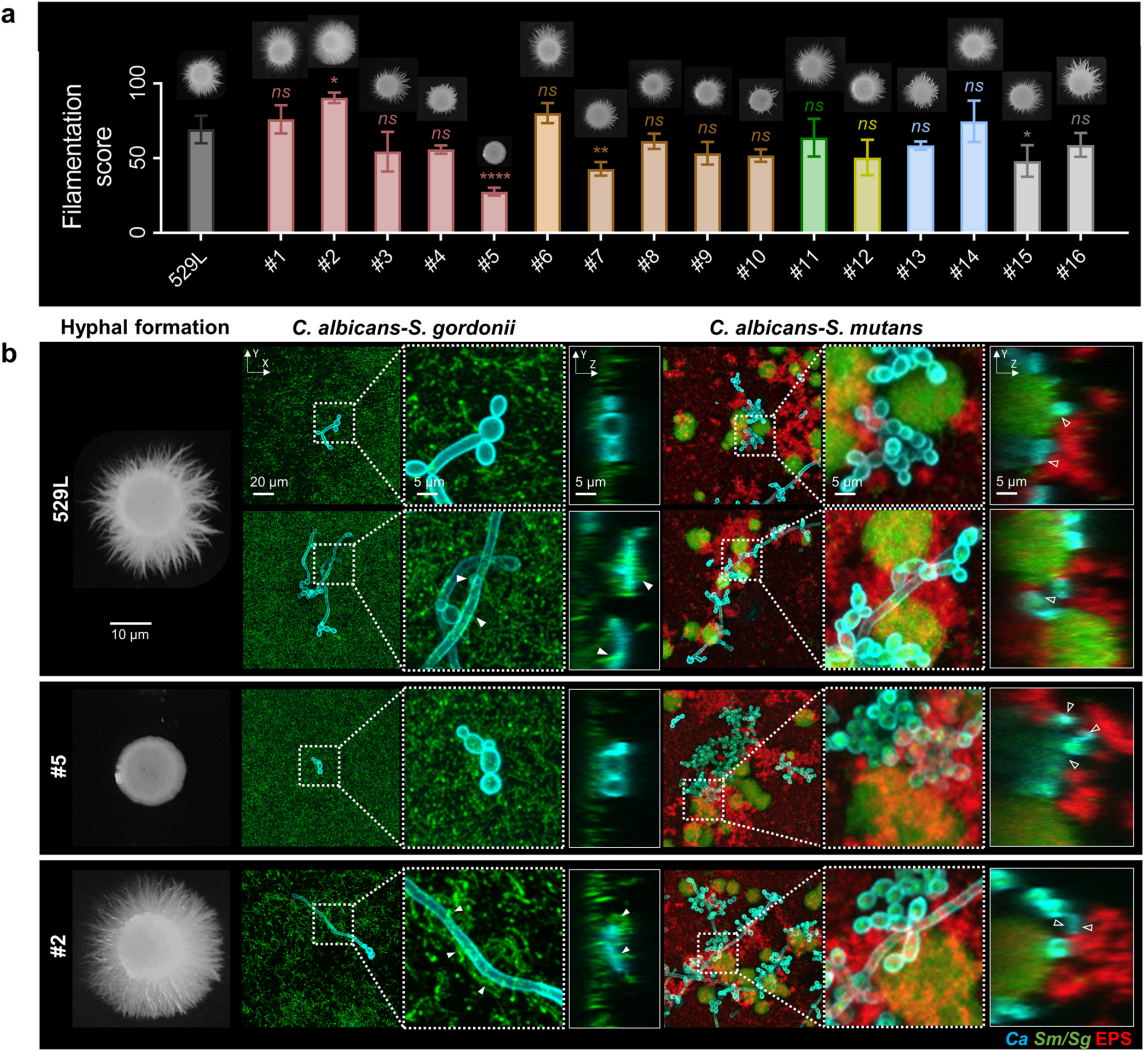
Hyphal formation capacity by C. albicans isolates and interkingdom interactions with commensal and cariogenic bacteria. (a) Hyphal formation of C. albicans isolates grown on Spider medium. Filamentation values are presented as mean ± SD of *n* = 3 independent replicates. Values are significantly different from 529L (reference strain) at *, *P < *0.05; **, *P < *0.01; ***, *P < *0.001; and ****, *P < *0.0001, while *ns* indicates that the values are not significantly different from 529L. (b) Representative confocal images of cell interactions of selected C. albicans isolates with commensal or cariogenic bacteria. Fungal cells were labeled with concanavalin A-tetramethylrhodamine (blue), bacterial cells with SYTO 9 (green), and EPS α-glucan with Alexa Fluor 647-dextran conjugate (red). White triangles indicate S. gordonii intertwined with C. albicans; hollow triangles indicate C. albicans cells (yeast form in isolate 5 and hyphae form in isolate 2).

C. albicans morphogenesis (yeast-to-hyphae transition) is considered a key factor for its virulence and biofilm formation in mucosal infections. Here, we used the established carbon-limited Spider medium method to assess hyphal formation by C. albicans isolates (32). The morphology of the colony formed on a Spider solid plate was scored based on central region and peripheral filamentation (23). The laboratory strain SC5314 served as a positive control, with the highest filamentation score of 100, while *efg1Δ/Δ*, which is defective in hyphae formation, served as a negative control and was given 0 for filamentation score (Fig. S2). Among all the tooth isolates, isolate 2 showed dense and long hyphae formation around the colony and had the highest score (Fig. 3a). In contrast, isolate 5 had the lowest score, with nearly no filamentation. Overall, C. albicans isolates varied broadly in terms of hyphal formation, which might influence interkingdom biofilm formation.

10.1128/mbio.02769-22.4 FIG S2 Reference strains for C. albicans hyphal formation on Spider medium. Download FIG S2, TIF file, 1.2 MB. Copyright © 2023 Xiang et al. 2023 Xiang et al. https://creativecommons.org/licenses/by/4.0/ This content is distributed under the terms of the Creative Commons Attribution 4.0 International license.

### Interkingdom interaction with commensal and cariogenic streptococci.

The interactions between C. albicans and bacteria are associated with enhanced virulence in oral diseases such as denture stomatitis and dental caries (33). During the interactions between C. albicans and mitis group streptococci, such as S. gordonii, the bacterium adheres to C. albicans hypha-specific adhesins via antigen I/II family polypeptides SspA/B (34). Therefore, hyphal formation is essential in exacerbating the severity of mucosal infections (4). However, the role of hyphal formation in interkingdom interactions with cariogenic species (S. mutans) remains unclear.

Considering the variation in fungal filamentation, we selected isolate 5 (hyphae defective), isolate 2 (hyperfilamentous), and 529L (reference) to further explore the interkingdom interactions with commensal or cariogenic streptococci. First, we assessed the initial stage (10 h) of interkingdom growth of C. albicans with S. gordonii or S. mutans by using high-resolution confocal imaging. As shown in Fig. 3b, S. gordonii cells bound and intertwined with isolate 2 (enhanced filamentation) through bacteria-hyphae association, whereas negligible interactions were observed with isolate 5 (devoid of hyphal forms), consistent with previous studies showing that S. gordonii specifically coadheres to the hyphal cell surface with high binding affinity (35). In contrast, C. albicans can develop interactions with S. mutans regardless of fungal morphology. Our data revealed that both yeast and hyphal forms of C. albicans 529L were associated with S. mutans cell clusters surrounded by the exopolysaccharide (EPS) matrix. Although the morphology of isolate 5 was altered due to lack of hyphal formation, the yeast clusters were bound to S. mutans cell clusters and enmeshed with abundant EPS (Fig. 3b), demonstrating capacity for interkingdom interactions despite the inability to form hyphae. Likewise, a similar association was observed between isolate 2 and S. mutans, although the spatial arrangement was noticeably distinctive, with hyphal forms that connected the yeast cells and S. mutans clusters.

### Biochemical interactions between C. albicans isolates and S. mutans.

**FIG 4:**
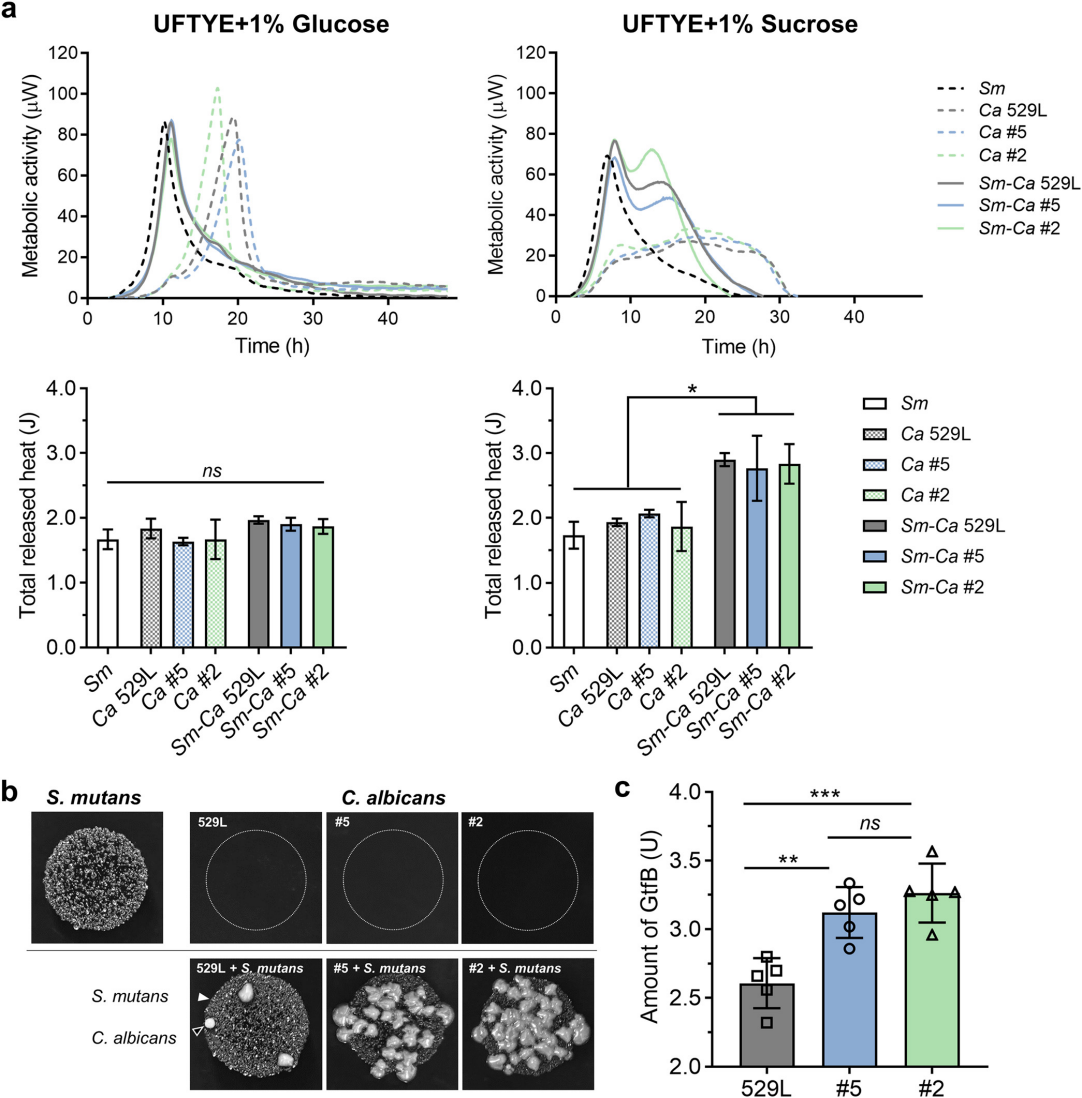
Symbiotic relationship between C. albicans strains and S. mutans promoted by metabolic and enzymatic interactions. (a) Isothermal microcalorimetry profiles of C. albicans strains in single- and dual-species growth with the presence of different carbon sources. (b) Colony-forming assay (hollow triangle indicates C. albicans, and white triangle indicates S. mutans) on mitis salivarius agar (MSA) containing 15% sucrose indicating cross-feeding of sucrose breakdown products for bacterial-fungal interaction. (c) Amount of S. mutans GtfB binding to C. albicans strains. Data are presented as mean ± SD of *n* = 3 (or *n* = 5 in c) independent replicates. Values are significantly different from each other at *, *P < *0.05; **, *P < *0.01; and ***, *P < *0.001, while *ns* indicates that the values are not significantly different from each other.

Previous studies indicated that the interkingdom relationship between C. albicans and S. mutans can be associated with sucrose metabolism (15, 17, 18). We next employed isothermal microcalorimetry (IMC) to measure the real-time metabolic activity of actively growing microbial cells to examine whether C. albicans isolates vary in the sugar metabolism of their interactions with S. mutans (36). The thermograms obtained by exposing C. albicans strains and/or S. mutans to different carbon sources (glucose and sucrose) in the planktonic phase are displayed in Fig. 4a. In the presence of glucose, C. albicans isolate 2 produced the highest peak and rate of metabolic activity and the fastest times to peak, while isolate 5 showed a lower peak metabolic rate with a longer time than 529L; however, the metabolic activity curves were similar when C. albicans strains were cocultured with S. mutans (Fig. 4a, top left panel). Despite different metabolic peak and rate patterns, mono- and cocultures growing in glucose showed no significant difference in cumulative (total) metabolic heat (area under the concentration-time curve [AUC]) (Fig. 4a, bottom left panel).

Under sucrose-supplemented conditions (Fig. 4a, top right panel), all C. albicans monocultures showed reduced metabolic activity compared to glucose conditions (Fig. 4a, top left panel). Remarkably, coculturing with S. mutans dramatically increased the metabolic activity, with two metabolic peaks compared to their monocultures (Fig. 4a, top right panel). The first peak appeared around 8 h after coculturing and overlapped with the peak of S. mutans monoculture, while the second peak, occurring after 10 h of incubation, aligned with the peaks of C. albicans monoculture under glucose conditions. Notably, the coculture of C. albicans isolate 2 and S. mutans produced the highest rate of metabolic activity at the second peak. Moreover, the total metabolic activity rendered by the cocultures was significantly higher than either C. albicans or S. mutans monococultures (Fig. 4a, bottom right panel), suggesting interkingdom synergism in carbohydrate metabolism.

We reasoned that C. albicans, which does not metabolize sucrose efficiently, may utilize the carbon source provided by S. mutans when they were cocultured since S. mutans can break down sucrose into fructose and glucose to promote cross-feeding (18, 37). To assess this possibility, we further tested the phenotype of C. albicans isolates grown either alone or with S. mutans in the presence of sucrose on agar plates (Fig. 4b and Fig. S3a). Most C. albicans isolates were unable to grow on sucrose-supplemented agar, except isolates 4 and 12, where very small colonies grew sparsely (Fig. S3a). In sharp contrast, when cocultured with S. mutans, all isolates were able to grow and form dense colonies juxtaposed with S. mutans. Notably, 529L showed many fewer colonies than tooth isolates 2 and 5 (Fig. 4b), suggesting a less effective cross-feeding interaction and sucrose metabolism.

10.1128/mbio.02769-22.5 FIG S3 Metabolic and enzymatic interactions between C. albicans strains and S. mutans. (a) Cross-feeding of sucrose breakdown products for symbiotic bacterial-fungal interaction. (b) Amount of S. mutans GtfB binding to C. albicans isolates. Values were presented as mean ± SD of *n* = 5 independent replicates. Download FIG S3, TIF file, 2.8 MB. Copyright © 2023 Xiang et al. 2023 Xiang et al. https://creativecommons.org/licenses/by/4.0/ This content is distributed under the terms of the Creative Commons Attribution 4.0 International license.

Another important factor in interkingdom interactions is the exoenzyme GtfB from S. mutans, which can bind to the cell wall surface of C. albicans in active form and synthesize glucans *in situ*, contributing to biofilm EPS matrix development (17). We determined the amount of GtfB bound onto the surface of C. albicans isolates using radiolabeling and scintillation counting (Fig. 4c and Fig. S3b). As shown in Fig. 4c, isolates 2 and 5 showed comparable amounts of surface-bound GtfB. However, the amounts of GtfB bound to C. albicans tooth isolates (2 and 5) were significantly higher than reference 529L, suggesting differences in the C. albicans cell wall composition/structure that mediate GtfB binding (17).

Altogether, C. albicans tooth isolates can develop interkingdom interactions with S. mutans irrespective of their ability to develop hyphae (a phenotypic trademark in C. albicans mucosal infections). Notably, tooth isolates showed enhanced metabolic activity, sucrose utilization with S. mutans, and GtfB binding compared to the reference oral mucosal strain, which can contribute to microbial growth, acidogenicity, and EPS matrix production to promote interkingdom biofilm development.

### Structure and acidogenicity of interkingdom biofilms.

**FIG 5:**
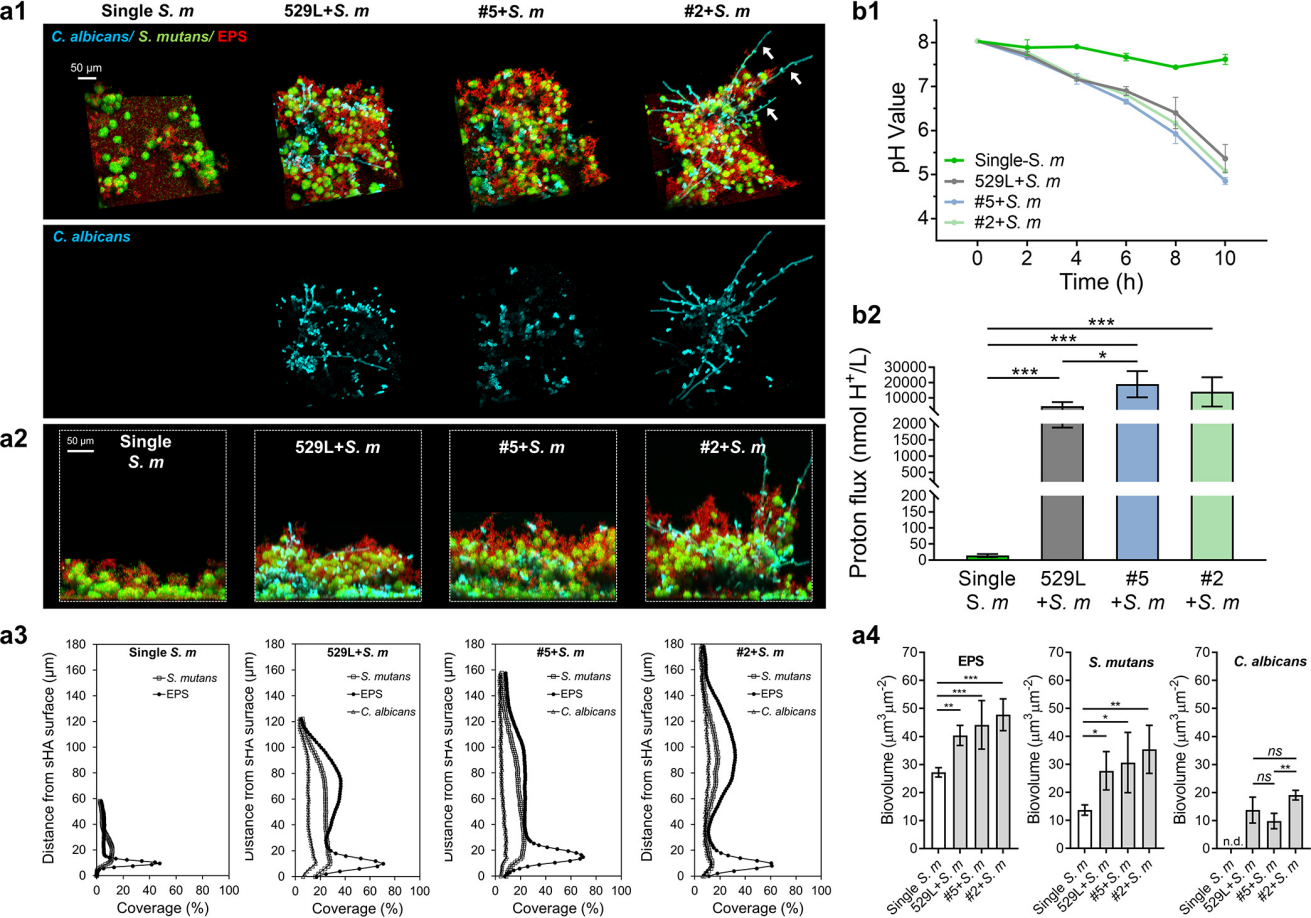
Three-dimensional (3D) architecture of cross-kingdom biofilm and saliva acidification. (a) Cross-kingdom biofilm formation (19 h) of C. albicans isolates and S. mutans. (a1) Representative images of three-dimensional architecture of S. mutans single-species biofilm or C. albicans and S. mutans dual-species biofilm. Fungal cells were labeled with concanavalin A-tetramethylrhodamine (blue), bacterial cells with SYTO 9 (green), and EPS α-glucan with Alexa Fluor 647-dextran conjugate (red). White arrows indicate hyphae extending throughout the biofilm architecture. (a2) Orthogonal views of the biofilm. (a3) Distribution of EPS, bacteria, and fungi in the biofilm (as performed with COMSTAT). (a4) Quantitative analysis of biovolume for each component in the biofilm (as performed with COMSTAT). (b) Biofilm acidification in saliva. (b1) Time-lapse pH measurements of biofilms. (b2) Proton flux rates in saliva during 4 to 10 h. Data were presented as nanomoles of protons per liter. Data are presented as mean ± SD of *n* = 5 independent replicates. Values are significantly different from each other at *, *P < *0.05; **, *P < *0.01; ***, *P < *0.001; and ****, *P < *0.0001 (a4 and b2), while *ns* indicates that the values are not significantly different from each other (a4).

To assess the influence of C. albicans tooth isolates in interkingdom biofilm formation, we analyzed the three-dimensional structure and the microbial spatial organization. We found that C. albicans tooth isolates significantly enhanced the thickness and biomass of the biofilms when grown together with S. mutans, resulting in increased accumulation of bacterial clusters and EPS, which were enmeshed with fungal cells (Fig. 5a, panels a1 and a2; Fig. S4). These findings were further confirmed by quantitative computational analyses of the biovolume of EPS and microbial components in relation to biofilm thickness (Fig. 5a, panels a3 and a4). The analyses revealed that the mixed species formed thicker biofilms than either S. mutans or C. albicans single-species biofilm counterparts (Fig. S5), with significantly enhanced biovolumes of EPS and bacterial cells. However, future studies using time-lapse imaging to monitor EPS synthesis in real time are needed to assess the dynamics of polymeric matrix assembly and spatial colocalization of EPS and C. albicans throughout the biofilm three-dimensional (3D) architecture.

10.1128/mbio.02769-22.6 FIG S4 Cross-kingdom biofilm (mature stage for 43 h) of C. albicans isolates and S. mutans. (a and d) Representative images of the three-dimensional architecture of single-species and dual-species biofilms. (b and e) Total viable counts (CFU) of S. mutans/C. albicans in single-species and dual-species biofilms. (c and f) Total amount of insoluble dry weight of single-species and dual-species biofilms. Data were presented as mean ± SD of *n* = 5 independent replicates. Values are significantly different from each other at **, *P < *0.01; ***, *P < *0.001; and ****, *P < *0.0001, while *ns* indicates that the values are not significantly different from each other. Download FIG S4, TIF file, 2.2 MB. Copyright © 2023 Xiang et al. 2023 Xiang et al. https://creativecommons.org/licenses/by/4.0/ This content is distributed under the terms of the Creative Commons Attribution 4.0 International license.

10.1128/mbio.02769-22.7 FIG S5 Three-dimensional architecture of single-species C. albicans biofilm. Download FIG S5, TIF file, 2.0 MB. Copyright © 2023 Xiang et al. 2023 Xiang et al. https://creativecommons.org/licenses/by/4.0/ This content is distributed under the terms of the Creative Commons Attribution 4.0 International license.

When comparing the different interkingdom biofilms, we noticed that 529L and isolates 5 and 2 exhibited robust accumulation of C. albicans cells intermixed with S. mutans clusters. However, each C. albicans strain developed structurally distinctive interkingdom biofilms. Within biofilm formed by isolate 5 (hyphae defective), the yeast cells accumulated around S. mutans clusters and were enmeshed in the EPS matrix. In contrast, noticeably more and longer hyphal forms were observed in biofilm formed by isolate 2 (hyperfilamentous) interspersed with S. mutans and EPS, extending throughout the biofilm architecture and even protruding outwards into the fluid phase (see white arrows), increasing the overall thickness. Conversely, the reference strain 529L developed biofilms containing both yeast and hyphal cells, showing an intermediary phenotype between isolates 2 and 5. Quantitatively, isolates 5 and 2 formed thicker biofilms (154 and 162 μm, respectively) than 529L (116 μm) (Fig. 5a, panel a3).

Next, we assessed the acidogenicity of interkingdom biofilms formed by C. albicans isolates and S. mutans in the presence of buffering saliva over time. S. mutans single-species biofilm slightly dropped the pH of saliva and stayed close to neutral (Fig. 5b, panel b1; pH 7.4 to 7.5). In contrast, interkingdom biofilms rapidly lowered the saliva pH after sucrose exposure and reached a value lower than the critical pH value (<pH 5.5) (Fig. 5b, panel b1). We also calculated the rate of proton accumulation, and the data showed a substantial increase in acid production in the interkingdom biofilm compared to S. mutans single-species biofilm (Fig. 5b, panel b2), consistent with the enhanced sugar cometabolism. Interestingly, we found that the interkingdom biofilm formed by 529L had a lower acid production rate than tooth isolates, congruent with reduced acid tolerance and less effective cross-feeding interaction in sucrose (versus clinical isolates). Our data show that interactions between C. albicans tooth isolates and S. mutans promote the development of interkingdom biofilm with enhanced acidogenicity regardless of fungal morphotypes. Furthermore, the tooth isolates form thicker biofilms with increased acidogenicity than oral mucosa strain 529L, suggesting that C. albicans in plaque exploits different pathways to survive and cause disease on the mineralized tissue.

### C. albicans transcriptional profiling in interkingdom biofilm with S. mutans.

**FIG 6:**
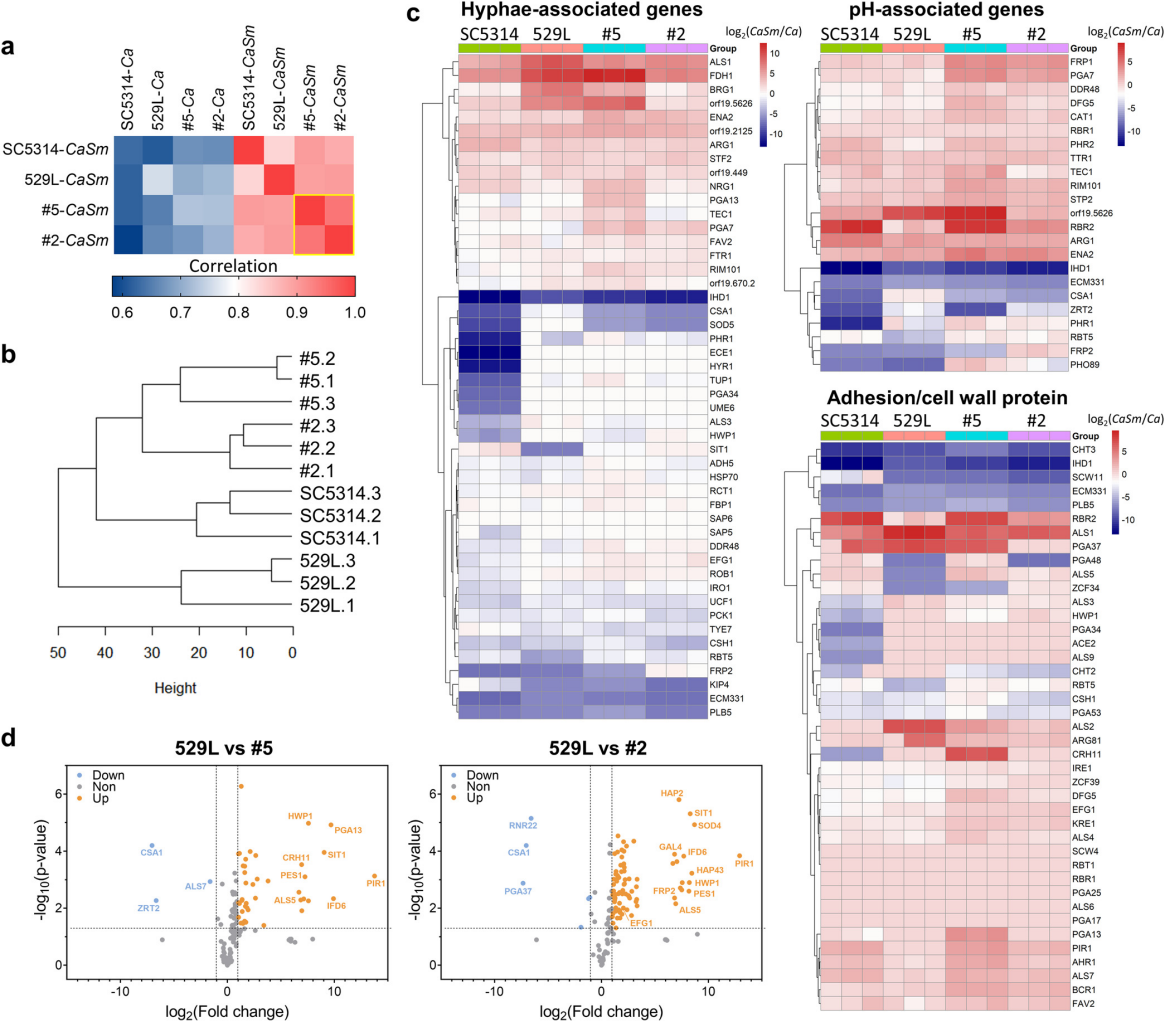
Transcriptional profiling of C. albicans strains in interkingdom biofilms with S. mutans. (a) The normalized RNA counts were analyzed for correlation using Spearman’s coefficient, and the correlation values were calculated in all pairwise combinations and visualized as a heatmap. Groups with strong clustering are outlined in yellow. (b) Hierarchical clustering of C. albicans strains. (c) Heatmap of differentially expressed genes in interkingdom biofilm compared to monospecies biofilm counterparts. The expression levels of hyphae-associated genes, pH-associated genes, and adhesion/cell wall protein-coding genes are shown. (d) Volcano plots showing the fold change and *P* value of upregulated (orange) and downregulated (blue) genes compared to 529L.

We next employed the NanoString nCounter to further investigate C. albicans tooth isolates in interkingdom biofilms at the transcriptional level (38) (Fig. 6 and Table S2). We first compared the gene expression profile across different C. albicans strains in monospecies or interkingdom biofilms. As shown in Fig. 6a, the absolute expressions of the tested genes were significantly correlated for all groups. The correlation between C. albicans gene expression in interkingdom biofilm and monospecies biofilm (left 4 columns) was much weaker than those between the same type of the biofilm (right 4 columns) (average, 0.670 versus 0.892; range, 0.611 to 0.754 versus 0.836 to 0.940, respectively), indicating differential transcriptome responses in C. albicans when interacting with S. mutans. Notably, the gene expression of isolates 2 and 5 showed the strongest correlation as outlined in yellow (Fig. 6a), suggesting the similarity of the transcriptional responses between tooth isolates during their interaction with S. mutans. The similarity was further supported by hierarchical clustering of transcripts values, showing that tooth isolates 5 and 2 were more closely related (Fig. 6b). Interestingly, 529L displayed the most divergent gene expression compared to other strains.

10.1128/mbio.02769-22.2 TABLE S2 Summary of gene expression profiles of C. albicans strains in monospecies or interkingdom biofilms. Raw RNA counts for each gene, normalized RNA counts, fold changes, and statistical significance of change (Student’s *t* test, *P < *0.05) are shown. The fold changes shown in orange are genes with statistically significant increased expression of >2-fold. Fold changes in blue are statistically significant reductions in gene expression of >2-fold. Download Table S2, XLSX file, 0.4 MB. Copyright © 2023 Xiang et al. 2023 Xiang et al. https://creativecommons.org/licenses/by/4.0/ This content is distributed under the terms of the Creative Commons Attribution 4.0 International license.

We then identified differentially expressed genes in interkingdom biofilm compared to monospecies biofilm counterparts, including those associated with filamentation, pH-related processes, and adhesion/cell wall structure. The effect of S. mutans on the transcriptional profile of the reference strain reveals insights into the nature of the bacterial-fungal interactions (Fig. 6c). The presence of S. mutans affects the expression of hyphae-associated genes, including hyphae-specific genes (*ALS3*, *HWP1*, *ALS1*, and *IHD1*), genes of virulence proteins secreted by hyphae (*ECE1*, *SAP6*), and master transcriptional regulators of hyphae and biofilm formation (*EFG1*, *ROB1*, and *UME6*), suggesting bacterial regulation of hyphal formation in interkingdom interactions. Furthermore, genes associated with pH-related processes and adhesion/cell wall protein were significantly enhanced in interkingdom biofilms (Fig. 6c). For example, *RIM101* and *PHR2*, which govern transcriptional responses to environmental pH, were found to be upregulated. Genes involved in cell wall synthesis, such as *PGA13* and *KRE1*, were also induced in interkingdom biofilms.

Compared with the reference mucosal strain (529L), 45 genes were differentially expressed between 529L and isolate 5, whereas 88 genes were differentially expressed between 529L and isolate 2 (*P < *0.05, fold change > 2; the color orange represents significantly upregulated and blue represents significantly downregulated genes) (Fig. 6d and Table S2). Interestingly, global repressors of hyphae formation, *TUP1* and its cofactor, *NRG1*, were significantly upregulated in the isolate 5 transcriptome compared to 529L and isolate 2, supporting its lack of filamentation. Remarkably, *PIR1*, which encodes C. albicans cell wall structural protein Pir1p, was one of the most upregulated genes, with a log_2_ fold change of >10 in both isolates 5 and 2. Pir1p is involved in the cross-linking of β-1,3-glucan that is bound to mannoproteins located on the outer surface of C. albicans, which are critical cell wall components mediating GtfB binding (17), which may explain the enhanced GtfB binding of the tooth isolates.

Altogether, these results further demonstrate that the tooth isolates are transcriptionally distinct with unique expression patterns associated with pH response and adhesion/cell wall structure compared to reference strains SC5314 and 529L.

## DISCUSSION

C. albicans has been found on the mineralized tooth surface and is associated with severe early childhood caries (S-ECC) (13, 39). How C. albicans adapts in biofilms on teeth and whether there are specific traits that contribute to biofilm virulence remain unclear. Our data show that C. albicans tooth isolates exhibit unique genotypic and phenotypic variations, consistent with C. albicans plasticity to adapt to different environments (23). Using C. albicans isolated from plaque of children with S-ECC, we find that hyphal formation, a hallmark for fungal virulence in mucosal infection, is not a phenotypic marker in tooth isolates. In contrast, all isolates display virulence attributes associated with caries pathogenesis. Three interesting features are conserved among the clinical isolates, which may have implications for the fungal ability to reside in plaque biofilms and cause disease in the tooth niche. We find that all C. albicans tooth isolates exhibit high proteinase activity and acidogenic and aciduric properties, despite their variations to other stress tolerance (oxidative, antifungal) traits and filamentation. The production of proteases such as Saps can contribute to caries severity, as these enzymes are capable of degrading dentinal collagen even under acidic conditions (40). Furthermore, C. albicans can produce short-chain carboxylic acids, such as pyruvates and formates (41), which help generate acidic biofilms. The ability of C. albicans tooth isolates to produce acids and grow under low-pH conditions, combined with heightened proteolytic activity, can promote enamel demineralization and further dentinal tissue destruction, which are the driving factors for the severe lesions found in S-ECC (27).

Another key finding is that the tooth isolates exhibit broad variations in hyphal growth, ranging from hyphae defective to hyperfilamentous. The switch from yeast to hyphae has been known to mediate C. albicans colonization, tissue destruction, and immune evasion in the host (42). Indeed, hyphae-mediated intraepithelial invasion and bacterial-fungal interactions are considered critical for oral and systemic candidiasis (43, 44). Mutants defective in hyphae formation or hyphal-specific adhesins are avirulent and unable to form biofilms with bacterial species, including mitis streptococci (e.g., S. gordonii) (34, 45, 46). However, interkingdom biofilms with S. mutans can be developed in the absence of hyphal forms (isolate 5), although superfilamentation (isolate 2) creates a distinctive spatial structure with exopolysaccharide (EPS)-coated hyphae protruding orthogonally. Notably, hyphae-defective (isolate 5) or hyperfilamentous (isolate 2) C. albicans isolates bind larger amounts of GtfB than the reference, 529L, suggesting compositional differences in the fungal membrane, as GtfB binds to the cell wall of both yeast and hyphae in active form to produce EPS α-glucans (17, 35, 47). This observation is consistent with biofilm experiments showing increased biomass and EPS accumulation in the interkingdom biofilms with either tooth isolate (versus 529L) or enhanced expression of *PIR1* that mediates the outer cell wall composition associated with GtfB binding.

The enhanced GtfB binding also influences metabolic activity and biofilm formation by C. albicans since the enzyme can release glucose during glucan synthesis, which enhances both fungal growth and acid production (18). Coculturing studies using hyphae-defective or hyperfilamentous C. albicans isolates reveal an interdependent relationship with S. mutans, which results in increased bacterial and fungal metabolic activity and growth in sucrose. Interestingly, tooth isolates form thicker and more metabolically active and acidogenic interkingdom biofilms that are capable of lowering the pH in saliva from neutral to below pH 5.5 (the pathological pH for tooth enamel demineralization) more rapidly than mucosal isolate 529L. Conversely, the presence of C. albicans also provides benefits to S. mutans in at least two ways. C. albicans secretes small amounts of farnesol that induces *gtfB* expression while promoting S. mutans growth (15). The GtfB bound onto the C. albicans cell surface converts sucrose to large amounts of α-glucans *in situ*, which, in turn, provide specific bacterial binding sites through S. mutans membrane-associated glucan binding proteins, promoting bacterial accumulation (33). Previous studies have also shown the enhanced ability of C. albicans and S. mutans to produce acids when growing together via a cross-feeding mechanism combined with elevated sugar cometabolism (16, 18). Due to increased growth and acid production by both species, interkingdom biofilms accumulate and lower the pH more effectively than either microbes alone, creating a highly cariogenic microenvironment.

C. albicans adopts distinct transcriptional profiles under different environmental conditions, which may contribute to its colonization and pathogenesis in different niches (48–50). Further analysis using NanoString reveals that tooth isolates display unique expression patterns compared to the reference strains SC5314 and 529L, particularly when forming biofilms with S. mutans. We find enhanced expression of C. albicans genes associated with pH responses, sugar metabolism, and adhesion/cell wall components within interkingdom biofilms, which are consistent with the increased biomass accumulation, metabolic activity, and acidogenicity. Notably, hyphae-defective and hyperfilamentous C. albicans tooth isolates show similar transcriptomic response in biofilms with S. mutans, but they are distinctive from those from SC5314 (bloodstream) and 529L (oral mucosal), suggesting niche-specific traits.

We recognize the complexity of bacterial-fungal association and the limitations of this study, including cross-sectional sampling, but also opportunities to motivate further investigations of C. albicans contributions to tooth niche. It should be noted that both hyphae-defective and hyperfilamentous phenotypes were observed in our growth conditions mimicking cariogenic biofilms *in vitro*, and further *in vivo* and genotyping analyses are needed to confirm the hyphal morphotypes. We are also employing time-lapse confocal imaging to investigate the spatiotemporal growth of C. albicans hyphae in the presence of S. mutans during biofilm initiation as well as their impact on enamel demineralization (51). The interactions between C. albicans and S. mutans are multifaceted and could induce additional responses in one another and/or alter the immediate environment to influence pathogenesis. Further characterization of tooth isolates obtained from longitudinal studies may reveal the dynamics of phenotypic/transcriptomic changes of C. albicans associated with the onset and severity of childhood caries. Studies on the fungal cell wall composition and structural organization between the mucosal strain and tooth isolates may provide additional insights into the differential GtfB binding affinity. Further in-depth analyses using *in vivo* models could elucidate the underlying mechanisms governing the tooth niche adaptation and interkingdom interactions as well as their impact on disease.

In conclusion, our findings provide new insights into C. albicans pathobiology to a nonmucosal human niche (tooth surface) where the fungal organism interacts with a bacterial pathogen (S. mutans) and contributes to biofilm virulence irrespective of different morphotypes, suggesting unique adaptive and interaction mechanisms in caries pathogenesis. Our study underlines the importance of understanding the behavior of C. albicans in distinct oral niches and how these differences affect virulence. Human tooth, a mineralized hard tissue, could provide additional knowledge of C. albicans colonization traits and mutualistic interkingdom interactions and help elucidate its role in the etiopathogenesis of S-ECC, which may lead to alternative therapeutic strategies targeting fungal contributions in an unresolved and costly pediatric disease.

## MATERIALS AND METHODS

### Microorganisms and growth conditions.

Supragingival plaque samples were collected from children (aged between 36 and 72 months) diagnosed with severe early childhood caries (S-ECC) as defined by the American Academy of Pediatric Dentistry. Ethical approval and written consent/permission forms of the study were obtained from the University of Pennsylvania’s Institutional Review Board (IRB no. 824243) prior to study commencement. The plaque samples were obtained from all available tooth surfaces with a sterile periodontal scaler and suspended in 1× phosphate-buffered saline (PBS) and transferred to the laboratory within 2 h. After collection, the plaque samples were gently vortexed and sonicated (three 10-s pulses with 30-s intervals at 7 W on ice; Branson Sonifier 150; Branson Ultrasonics, Danbury, CT, USA) followed by plating onto BBL CHROMagar *Candida* (BD, Sparks, MD, USA). The plates were incubated at 37°C for 48 h to isolate Candida albicans. Pure cultures of C. albicans were obtained based on colony color and morphology for presumptive identification (52). C. albicans identification was confirmed using colony PCR that allows the differentiation from non-*albicans Candida* species. The C. albicans tooth isolates (UR5, UR8, UR9, UR12, UR13, UR15, and UR18) were provided by Jin Xiao, University of Rochester, as previously reported in reference 9.

C. albicans SC5314 (a well-characterized laboratory strain), 529L (clinical strain isolated from oral candidiasis) (21), and hyphae-deficient strain SN250 *efg1Δ/Δ* served as reference strains. Streptococcus mutans UA159 serotype c (ATCC 700610), an established virulent cariogenic pathogen, and S. gordonii DL1, a commensal colonizer of dental biofilm that interacts with C. albicans on oral mucosal surfaces, were used in the present study. All the microorganisms were grown to mid-exponential phase in ultrafiltered (10-kDa-molecular-mass-cutoff membrane; Millipore, MA, USA) tryptone-yeast extract broth containing 2.5% tryptone and 1.5% yeast extract (UFTYE; pH 5.5 for C. albicans and pH 7.0 for S. mutans and S. gordonii) with 1% (wt/vol) glucose (37°C, 5% CO_2_) for inoculum preparation.

### Multilocus sequence typing.

MLST of C. albicans strains was performed by amplifying and sequencing seven C. albicans housekeeping genes (*AAT1a*, *ACC1*, *ADP1*, *MPIb*, *SYA1*, *VPS13*, and *ZWF1b*) as described previously (25). The distinct alleles of seven gene loci were identified and assigned to genotypes by reference to the MLST C. albicans database (https://pubmlst.org/organisms/candida-albicans). A diploid sequence type (DST) of each strain was also characterized by defining unique combinations of genotypes. To evaluate the genetic relatedness between the investigated strains, DNA sequences were concatenated, and a dendrogram was constructed based on the unweighted pair group method by using average linkages for cluster analysis and clade definition using MEGA software (http://www.megasoftware.net/), as described in reference 25.

### Phenotypic profiling assays.

**(i) Growth experiments.** Mid-exponential-phase cultures of each C. albicans strain were subcultured into UFTYE (pH 7.0 for planktonic growth and pH 4.0 for acid tolerance) and incubated at 37°C with 5% CO_2_. The optical density of the cell culture at 600 nm (OD_600_) was measured hourly using a spectrophotometer (Spectronic 20D+, Thermo Fisher Scientific Inc, Madison, WI). The growth curve was plotted by the OD_600_ against time.

**(ii) Spot assays for susceptibility testing.** The C. albicans cells were collected at mid-exponential phase and 10-fold serially diluted. The serial dilutions (10^2^, 10^3^, 10^4^, or 10^5^ CFU per spot) of each C. albicans culture were spotted onto yeast extract-peptone-dextrose (YPD) agar plates for serial susceptibility testing. For the regular growth test, YPD plates were incubated for 72 h at 37°C. For the heat stress tolerance test, the plates were subjected to 42°C incubation. For the oxidative stress test, hydrogen peroxide was added to the YPD medium at a final concentration of 10 mM. For the antifungal susceptibility test, the fungal cells were inoculated on YPD agar containing fluconazole at a concentration of 2 μg/mL. Growth differences were recorded following incubation at different conditions (53).

**(iii) Glycolytic pH drop assay.** The Acidogenicity of C. albicans strains was measured by glycolytic pH drop assay as described previously (54). The fungal cells were collected at mid-exponential phase and washed with salt solution (50 mM KCl, 1 mM MgCl_2_, pH 7.0). Glucose was added into the solution at a final concentration of 1% (wt/vol), which allows for the production of acids over time. The pH was measured using a pH electrode (Thermo Scientific, Waltham, MA, USA) every 30 min over a period of 120 min.

**(iv) Proteinase assay.** The enzymatic activity of secreted aspartyl proteinase (Sap) was tested according to the methodology as previously described with some modifications (31). The test medium consisted of agar plates containing 2 g bovine serum albumin (BSA), 20 g of dextrose, 1 g of K_2_HPO_4_, 0.5 g of MgSO_4_, and 15 g of agar in 1 L of distilled water. Briefly, the fungal cell suspension was adjusted to 10^6^ CFU/mL, and the suspension at 5 μL was inoculated onto the BSA plate and incubated at 37°C for 5 days. The diameters of colonies and colonies plus the zone of precipitation were measured. The proteinase activity (*Pz*) was determined by the ratio of colony diameter to colony-and-halo diameter. *Pz* coefficients were classified as negative (*Pz *= 1.00), positive (0.64 ≤* Pz *< 1.00), and strongly positive (*Pz *< 0.64).

**(v) Filamentation assay.** Spider agar plates (1% Difco nutrient broth, 1% mannitol, 0.2% dibasic potassium phosphate, and 1.5% agar, pH 7.2) were used to conduct morphogenesis screening of C. albicans strains (23). Briefly, 5 μL of each fungal cell suspension was dropped onto Spider agar at a density of 10^6^ CFU/mL. Colonies were photographed after incubation of 72 h, and filamentation scores were based on colony appearance. SC5314 served as a positive control, and hyphae-deficient strain *efg1*Δ/Δ was included as a negative control.

**(vi) Sucrose assimilation test.** Mitis salivarius agar (MSA) containing 15% sucrose was used to test the cross-feeding effect of sucrose breakdown products between C. albicans isolates and S. mutans. The C. albicans and S. mutans cells were collected at mid-exponential phase and adjusted to 10^4^ CFU/mL and 10^4^ CFU/mL, respectively. We spotted 5 μL of each cell suspension or the mixture of each C. albicans strain with S. mutans onto MSA plates. The plates were then incubated for 72 h and observed for growth.

### Isothermal microcalorimetry.

Metabolic profiles were measured using real-time isothermal microcalorimetry. The cultures of S. mutans and C. albicans were diluted to 10^6^ CFU/mL and 10^4^ CFU/mL, respectively, using fresh UFTYE medium. The inoculum sizes were used throughout the experiments to evaluate the metabolic profiles of mono- or cocultures of S. mutans and C. albicans under sucrose or glucose conditions. We transferred 200 μL of each microbial suspension in triplicate to sterile plastic inserts designed to fit in titanium measurement vials (calWell; SymCel Sverige AB, Stockholm, Sweden). The inserts were placed in the calWells, sealed, and loaded in the instrument calScreener microcalorimeter (SymCel Sverige AB). Real-time heat production and proxying metabolic activity measurements were performed using calScreener microcalorimeter with its corresponding 48-well plate (calPlate) as previously described (36). The microcalorimeter data were collected and quantified with calView software (version 1.0.33.0; SymCel Sverige AB). During the assays, the instrument was set and calibrated at 37°C. General handling and device manipulation were done according to the manufacturer’s recommendations.

### GtfB binding to the C. albicans cell surface.

GtfB was adsorbed to the microbial cells, and bound GtfB was determined by radiolabeling and scintillation counting as detailed in reference 47. Briefly, the GtfB enzyme was prepared from culture supernatants of Streptococcus milleri KSB8 constructs and purified by hydroxylapatite column chromatography. C. albicans cells (~2 × 10^7^ CFU/mL) were mixed with 25 μg/mL of GtfB in adsorption buffer and incubated for 60 min at 37°C with rocking as optimized previously (47). After incubation, the fungal cells were pelleted by centrifugation (6,000 × *g*, 10 min at 4°C) and washed three times with adsorption buffer to remove the loosely bound GtfB. Then, GtfB-coated yeast cells were resuspended in 250 μL of adsorption buffer and mixed with the same volume of [^14^C]glucose-sucrose (NEN Research Products, Boston, MA) substrate (0.2 μCi/mL; 100 mmol of sucrose/L, 20 μmol of dextran 9,000/L, and 0.02% sodium azide in adsorption buffer, pH 6.5) for 2 h at 37°C with rocking. The amount of enzymatic activity of surface-bound GtfB was measured by the incorporation of [^14^C]glucose from labeled sucrose into glucans using scintillation counting. One unit of enzyme activity is defined as the amount of GtfB enzyme that incorporates 1 μmol of glucose into glucans over the 2-h reaction. All assays were done in triplicate in at least three different experiments.

### Experimental biofilm model and quantitative biofilm analysis.

Biofilms were formed on saliva-coated hydroxyapatite (sHA) discs as described previously (55). Briefly, sHA discs (surface area, 2.7 ± 0.2 cm^2^; Clarkson Chromatography Products, Inc., South Williamsport, PA) were vertically suspended in 24-well plates using a custom-made wire disc holder, mimicking the smooth surfaces of the pellicle-coated teeth. Each sHA disc was inoculated with approximately 2 × 10^6^ CFU/mL of S. mutans or 2 × 10^6^ CFU/mL of S. gordonii and/or 2 × 10^4^ CFU/mL of C. albicans in UFTYE (pH 7.0) containing 1% (wt/vol) sucrose at 37°C with 5% CO_2_; the inoculated microorganisms share a similar proportion to that found in saliva samples from children with ECC (56). The culture medium was changed twice daily (at 19 and 29 h) until the end of the experimental period (43 h). The biofilms were collected and analyzed for biochemical and structural analysis as described below.

The 3D architecture of biofilm was assessed using confocal laser scanning microscopy (CLSM) combined with quantitative computational analysis as described elsewhere (17). Briefly, S. mutans cells (or S. gordonii cells) were stained with 2.5 μM SYTO 9 green fluorescent nucleic acid stain (485/498 nm; Molecular Probes Inc., Eugene, OR, USA), and C. albicans cells were stained using concanavalin A (ConA) lectin conjugated with tetramethylrhodamine at 40 μg/mL (555/580 nm; Molecular Probes Inc.), while EPS glucans were labeled via incorporation of 1 μM Alexa Fluor 647-dextran conjugate (647/668 nm; Molecular Probes Inc.) during biofilm formation. The biofilm imaging was performed using an upright single-photon confocal microscope (LSM800; Zeiss, Jena, Germany) equipped with a 20× (numerical aperture, 1.0) water immersion objective. Each component was illuminated sequentially to minimize cross talk as follows: SYTO 9 was excited at 488 nm and collected by a 480/40-nm emission filter, ConA was excited at 560 nm and collected by a 560/40-nm emission filter, and Alexa Fluor 647 was excited at 640 nm and collected by a 670/40-nm emission filter. The confocal images were acquired from at least 3 randomly selected spots of each sample. ImageJ software (version 1.48) was used to create 3D renderings of biofilm architecture, and COMSTAT was used for biomass quantification.

For microbiological and biochemical analyses, the biofilms were removed from sHA and homogenized via sonication, providing optimum dispersal and maximum recoverable counts without killing microbial cells. The homogenized suspension was used to determine the total number of viable cells in each of the biofilms. The remaining biofilm suspension was washed twice with Milli-Q water, oven-dried for 2 h, and weighed to obtain biomass as detailed previously (57).

To monitor real-time extracellular pH gradients, biofilms were grown to 19 h in UFTYE and transferred to filtered saliva supplemented with 1% (wt/vol) sucrose. The pH was measured every 2 h during the middle phase of biofilm culture (19 to 29 h) using a pH electrode (Thermo Scientific, Waltham, MA, USA). The proton accumulation was then calculated from the pH decrease.

### NanoString multiplexed analysis.

Biofilms were harvested after 19 h of incubation and subjected to RNA extraction (58, 59). Briefly, the disk sets were immersed in RNAlater (Applied Biosystems/Ambion, Austin, TX, USA), and then the biomass was removed from the sHA disks. Three independent biological replicates were collected, and the cells were centrifuged for 2 min at 11,000 rpm, followed by RNA extraction from the pellet according to the protocol of the manufacturer (MasterPure yeast RNA purification kit; catalog no. MPY03100; Epicentre Biotechnologies, Madison, WI). The NanoString nCounter analysis system was then used as previously described (59). Briefly, the purified RNA (40 ng/μL total RNA) was added to a NanoString CodeSet mix and incubated at 65°C for 18 h. After the hybridization reaction, samples were loaded onto nCounter prep station and scanned on an nCounter digital analyzer. The resulting nCounter RCC files for each sample were then imported into nSolver software to evaluate the quality control metrics. Using the negative-control probes, the background values were first assessed. The mean plus standard deviation of the negative-control probe value was defined and used as a background threshold, which was further subtracted from the raw counts. The background-subtracted total raw RNA counts were normalized against the highest total counts from the biological triplicates. The statistical significance of changes in counts was determined (Student’s *t* test, *P < *0.05), and the normalized expression data were summarized in Table S2. Probes that were below background were set to a value of 1 to allow statistical analysis. Further data visualization was conducted in R v4.1.2, and figures were made with the package ggPlot.

### Statistical analysis.

The results were expressed as mean ± standard deviation. Data were subjected to Student’s *t* test or analysis of variance (ANOVA) with *post hoc* Dunnett’s test for multiple comparisons. The significance levels were set at 0.05. The statistical analyses were carried out using GraphPad Prism 9 (GraphPad Software, San Diego, CA).
