# Supplementary figures and images for: Human Tooth as a Fungal Niche: Candida albicans Traits in Dental Plaque Isolates
Source: mBio. 2023 Jan 5;14(1):e02769-22. doi: 10.1128/mbio.02769-22 (PMC9973264; doi:10.1128/mbio.02769-22)

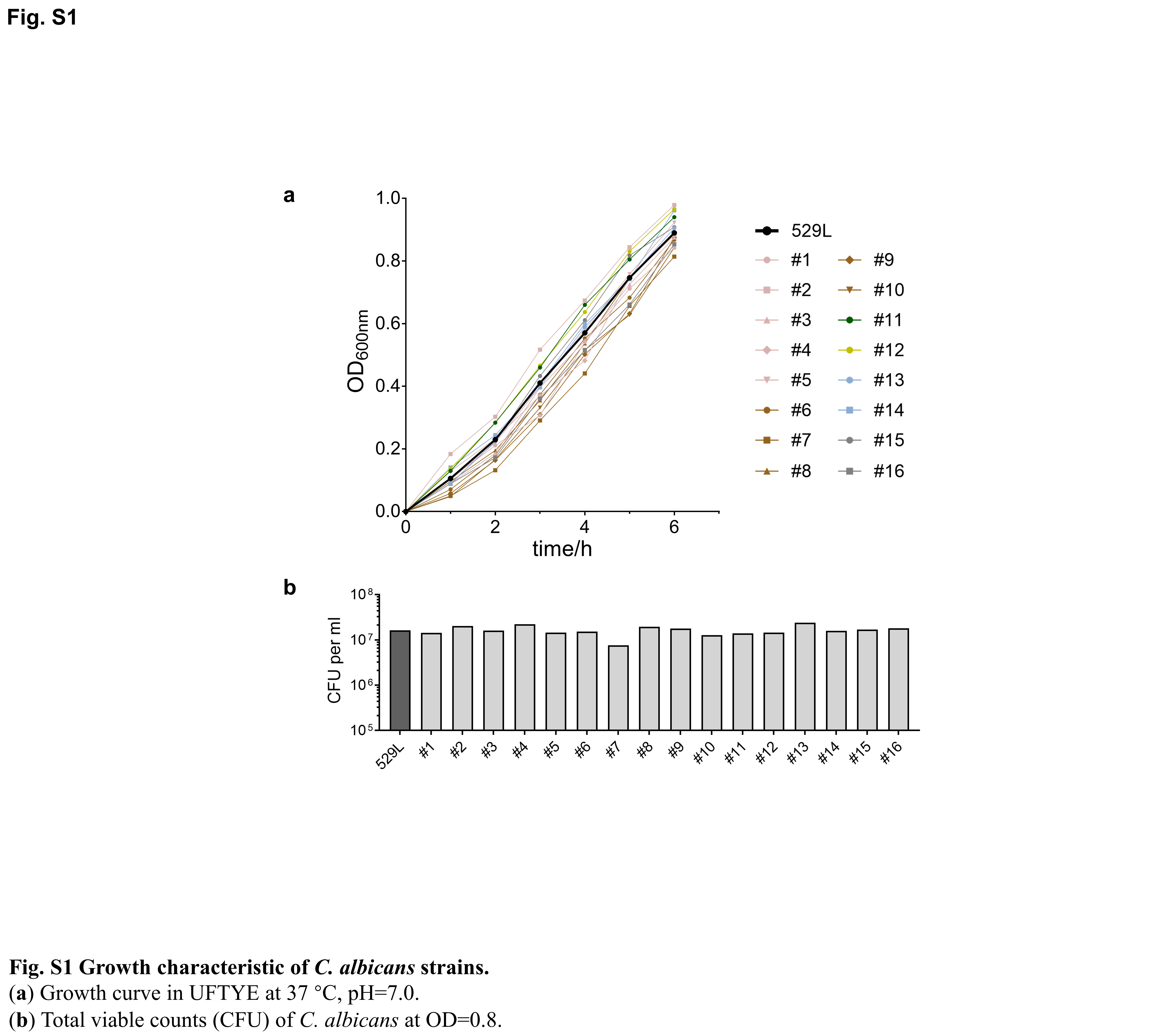

Supplement: FIG S1 [file mbio.02769-22-s0003.tif]

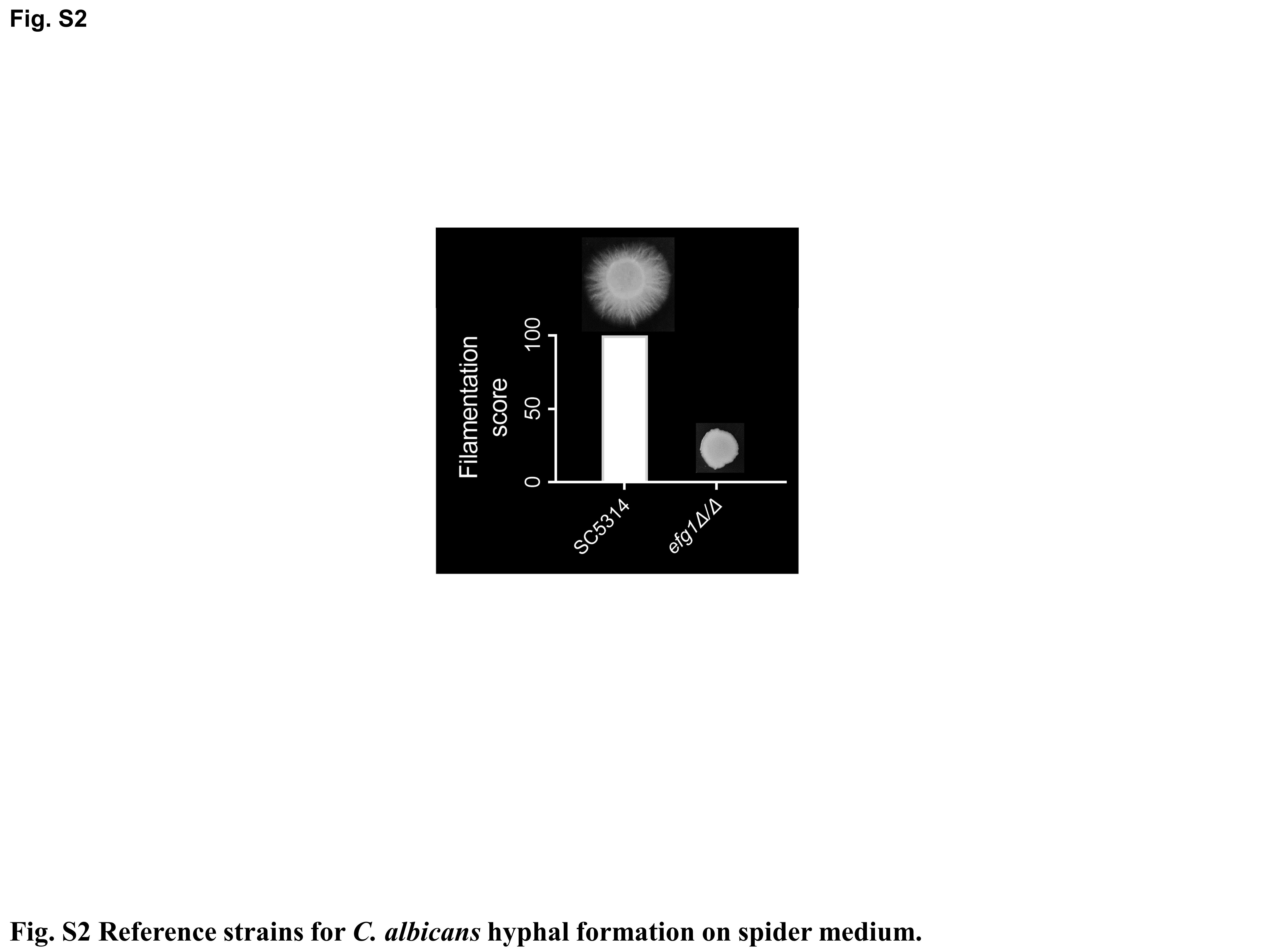

Supplement: FIG S2 [file mbio.02769-22-s0004.tif]

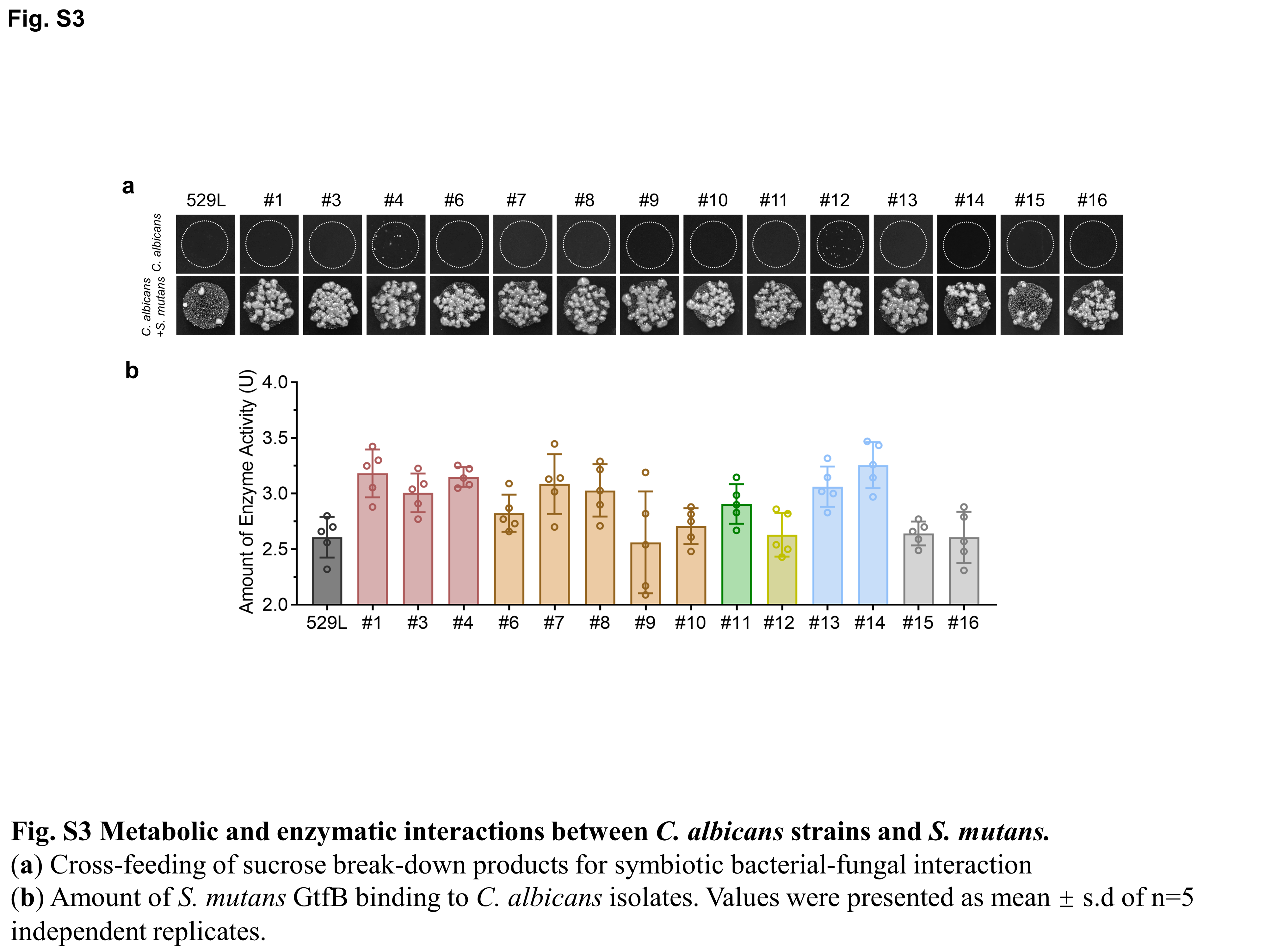

Supplement: FIG S3 [file mbio.02769-22-s0005.tif]

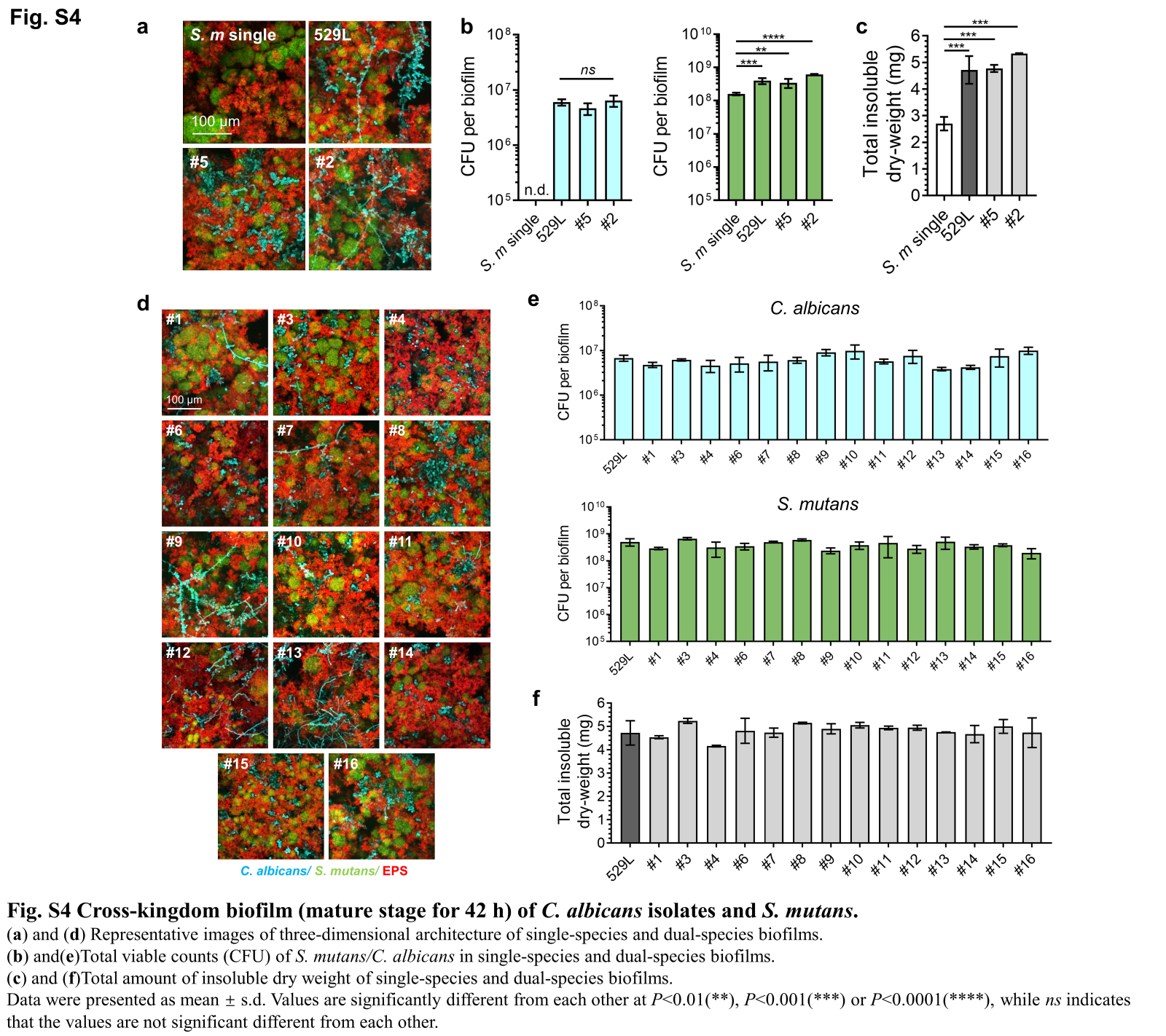

Supplement: FIG S4 [file mbio.02769-22-s0006.tif]

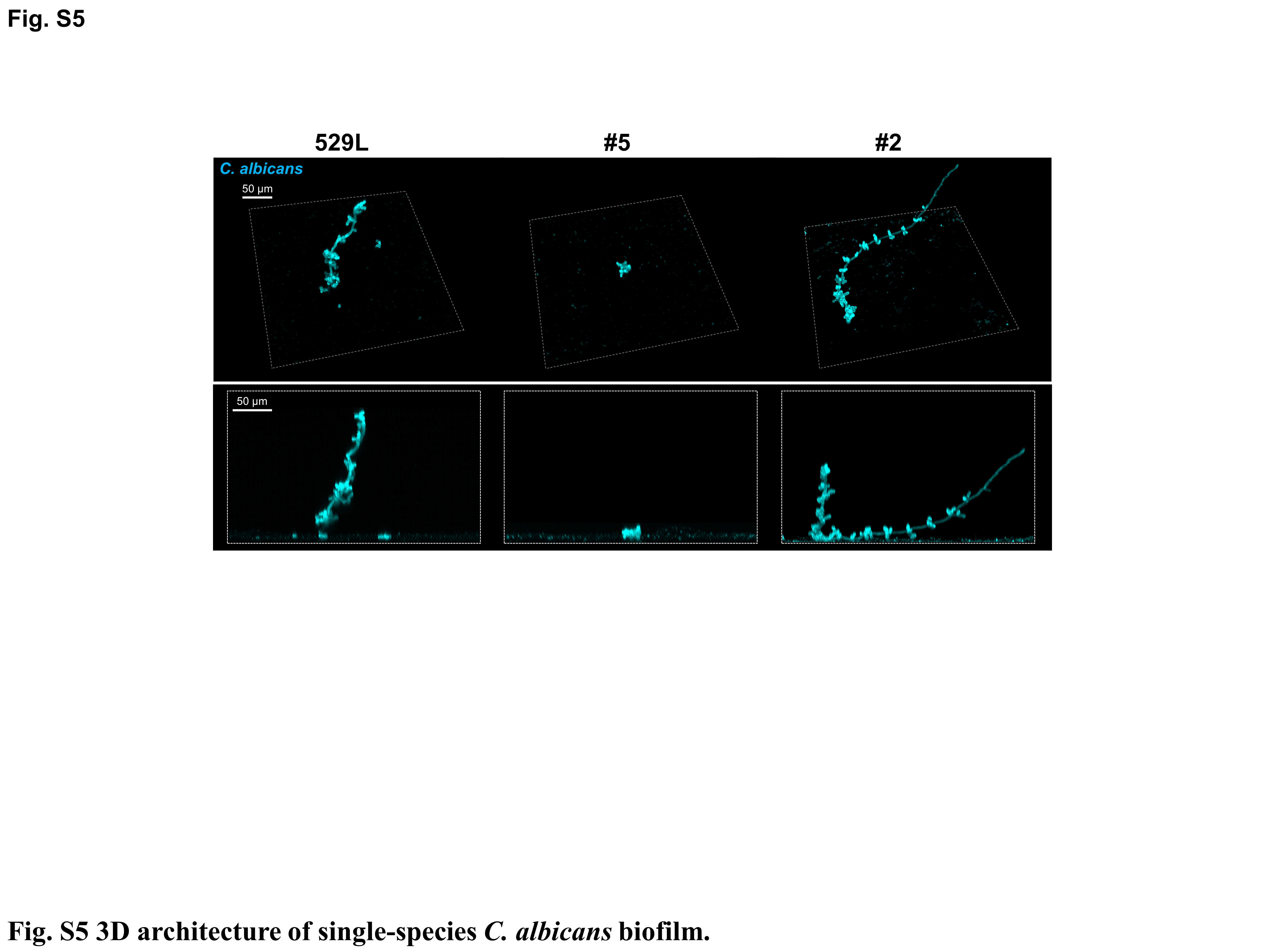

Supplement: FIG S5 [file mbio.02769-22-s0007.tif]
